# Hydrodynamic Regulation of Monocyte Inflammatory Response to an Intracellular Pathogen

**DOI:** 10.1371/journal.pone.0014492

**Published:** 2011-01-07

**Authors:** Shankar J. Evani, Ashlesh K. Murthy, Naresh Mareedu, Robbie K. Montgomery, Bernard P. Arulanandam, Anand K. Ramasubramanian

**Affiliations:** 1 Department of Biomedical Engineering, The University of Texas at San Antonio, San Antonio, Texas, United States of America; 2 Department of Biology, The University of Texas at San Antonio, San Antonio, Texas, United States of America; 3 South Texas Center for Emerging Infectious Diseases, The University of Texas at San Antonio, San Antonio, Texas, United States of America; Massachusetts General Hospital and Harvard Medical School, United States of America

## Abstract

Systemic bacterial infections elicit inflammatory response that promotes acute or chronic complications such as sepsis, arthritis or atherosclerosis. Of interest, cells in circulation experience hydrodynamic shear forces, which have been shown to be a potent regulator of cellular function in the vasculature and play an important role in maintaining tissue homeostasis. In this study, we have examined the effect of shear forces due to blood flow in modulating the inflammatory response of cells to infection. Using an *in vitro* model, we analyzed the effects of physiological levels of shear stress on the inflammatory response of monocytes infected with chlamydia, an intracellular pathogen which causes bronchitis and is implicated in the development of atherosclerosis. We found that chlamydial infection alters the morphology of monocytes and trigger the release of pro-inflammatory cytokines TNF-α, IL-8, IL-1β and IL-6. We also found that the exposure of chlamydia-infected monocytes to short durations of arterial shear stress significantly enhances the secretion of cytokines in a time-dependent manner and the expression of surface adhesion molecule ICAM-1. As a functional consequence, infection and shear stress increased monocyte adhesion to endothelial cells under flow and in the activation and aggregation of platelets. Overall, our study demonstrates that shear stress enhances the inflammatory response of monocytes to infection, suggesting that mechanical forces may contribute to disease pathophysiology. These results provide a novel perspective on our understanding of systemic infection and inflammation.

## Introduction

A number of opportunistic pathogenic organisms including bacteria and fungi can occasionally gain entry into the human cardiovascular system, resulting in acute or chronic complications. Systemic bacterial infections of *Staphylococcus* and *Streptococcus spp.*, *Porphyromonas gingivalis* or *Pseudomonas aeruginosa* are associated with acute bacteremia [1]. If left untreated, such acute infections can lead to life-threatening infective endocarditis, disseminated intravascular coagulation or immune thrombocytopenia [2]. In other instances, systemic bacterial infections are reported to play an important role in the progression of chronic diseases including coronary artery disease [3], [4].

Of interest, local hemodynamics and mechanical forces are critical in tissue homeostasis. [5], [6]. Fluid shear stress acting on cells in circulation stimulates cell signal transduction, gene expression, and affects cell shape and survival [7], [8], [9]. Such modulation of cellular function by biomechanical forces forms the basis of pathophysiology of vascular diseases such as atherosclerosis, wherein disturbed blood flow at arterial bends and branches favor a pro-atherogenic endothelium [10]. Laminar shear stress in bulk circulation also activates platelets and leukocytes to different levels [11], [12]. Collectively, these observations indicate that mechanical force due to blood flow may be an important determinant in the pathophysiology of systemic inflammation.

In this study, we have characterized the interaction of fluid shear stress on monocytes that are infected with an intracellular pathogen, Chlamydia. *Chlamydiae* are obligate intracellular pathogens that can survive and multiply only within host cells. An initial infection occurs following the entry of an infectious elementary body into host cells. Upon internalization, the elementary body (EB) differentiates into a noninfectious but metabolically active reticulate body (RB), which multiplies and subsequently differentiates back to an EB. During this cycle, mature EBs are released extracellularly and spread to other susceptible host cells. Chlamydial infection prevents apoptosis of host cells thus providing a safe haven for the bacterial growth. They are responsible for a wide array of diseases including pneumonia, bronchitis, infertility and pelvic inflammatory diseases [13], [14]. In particular, multiple lines of evidence implicate that *Chlamydia pneumoniae* infection is a highly likely risk factor for atherosclerosis, based on several *in vitro*, seroepidemiological, histopathological, animal models, and limited clinical intervention studies [15], [16]. *C. pneumoniae* infects neutrophils and alveolar macrophages, and is disseminated from the lung into the vasculature through peripheral blood mononuclear cells (PBMC) [17]. *In vivo*, chlamydial infection of circulating monocytes has been shown to last for at least up to seven days. During this period, infected monocytes secrete inflammatory cytokines, procoagulants, matrix metalloproteinases, and upregulate the expression of endothelial adhesion molecules, and LDL-uptake receptors.

Taken together, we hypothesized that fluid shear stress modulates the biochemical effects of chlamydial infection, and hence will alter the course of vascular inflammation. In order to test this hypothesis, we used an *in vitro* model employing THP-1 monocytic cell line infected with *Chlamydia muridarum*, and analyzed the effects of infection under well-controlled, physiologically relevant shear conditions. The THP-1 is an established cell line derived from a child with acute monocytic leukemia. This cell line is blocked at a relatively late stage of normal differentiation, and is committed to differentiate *in vitro* into native macrophage-like cells in the presence of activating signals [18]. THP-1 has been widely used as a reliable, fairly accurate approximation for human monocytes. *Chlamydia muridarum* infects and activates dendritic cells [19], macrophages [20], and neutrophils in the lung and urogenital tract. We have used *C. muridarum* as a model in order to capture the essential aspects of an intracellular infection. *C. muridarum* has a shorter development cycle (∼27 h), and has previously been employed in several studies to understand cellular and molecular aspects of chlamydial infection of human cells [21], [22], [23], and pathogenesis and host response to *C. pneumoniae* infection [24], [25], [26].

We characterized the effect of fluid shear stress on the inflammatory response of chlamydia-infected monocytes. We observed that chlamydial infection results in morphological changes in monocytes. The infected, but not uninfected monocytes, when subjected to short duration of physiological levels of shear stress, expressed significantly more chemokines and cytokines in a time-dependent fashion, compared to static conditions. We also observed that shear stress considerably enhanced the cell–cell interactions of vascular cells such as monocytes, platelets and endothelial cells. Taken together, we demonstrate that infection and shear stress act synergistically in heightening vascular inflammatory response. Most of our current understanding of blood stream infections is based on static assays, though static assays may not mimic the dynamic fluid mechanical environment of the vasculature [27]. Thus, our approach and results may have important implications in our understanding of blood stream infections and related diseases.

## Materials and Methods

### Ethics Statement

Venous blood was freshly drawn from healthy volunteers after signing an informed consent and obtaining written ethics approval in accordance with the Institutional Review Board (IRB) protocol (IRB #09-066, Office of Research Integrity and Compliance, UTSA).

### Bacteria

*Chlamydia muridarum* was grown in confluent monolayer of HeLa cells (ATCC) in DMEM (Cellgro) supplemented with sodium pyruvate and L-glutamine, and 10% FBS, gentamicin, and cycloheximide. 27 hours post infection, HeLa cells were harvested and lysed using a sonicator (Fisher). The elementary bodies (EB) were purified on Renograffin gradients, aliquoted in sucrose-phosphate-glutamine buffer and stored at −80°C as described [28]. *Chlamydia* genus-specific murine monoclonal antibody (Meridian Life Sciences) was used to establish the bacterial counts in stocks.

### Cells

Human monocyte cell line, THP-1 (ATCC) was cultured in RPMI 1640 (ATCC) supplemented with 10% FBS and .05 mM mercaptoethanol (Sigma), at 37°C and 5% CO_2_. The cells were passaged into fresh media when the cells reached a density of 10^6^/ml. Human Aortic Endothelial cells (HAEC) were obtained from Life Cell Technologies. The cells were originally extracted from the aorta of cadaver of an individual with no known cardiovascular abnormalities. The HAEC were cultured in media supplemented with 5% FBS as per the manufacturer's instructions.

### Platelets

The blood was collected in tubes containing acid-citrate-dextrose (ACD) (BD Biosciences). The blood was centrifuged at 250×g for 25 min at 24°C, to obtain platelet rich plasma (PRP) [29]. The platelet count was measured using a Coulter counter.

### Chlamydial infection on THP-1 cells

THP-1 cells were seeded in 35 mm culture dishes at a cell number of 3×10^6^/dish and infected with 6×10^6^ inclusion forming units (IFU) of chlamydial EB in a total volume of 600 µl/dish (Multiplicity of Infection, MOI 2). The dishes were incubated for 2 hours at 37°C in a humidified 5% CO_2_ incubator with intermittent shaking for every 15 minutes to ensure efficient infection. After 2 hours post infection, 2400 µl of RPMI complete supplemented with 1 µg/ml gentamicin (MP Biomedicals) was added to each dish, and returned to the cell culture incubator. 24 hours post infection, the cells were spun down and fixed in 2% freshly-prepared formaldehyde (Sigma) in PBS for 1 hour. The cell concentration was then adjusted to 5×10^5^/ml using PBS. These cells were cytospun on glass slides at 1000 RPM for 5 minutes with low acceleration. The slides were then blocked with 2% BSA, and stained with *Chlamydia* genus-specific murine monoclonal primary antibody and a FITC-conjugated rabbit anti-mouse secondary antibody to establish infectivity, and Hoechst nuclear counter-stain. Uninfected THP-1 cells (treated with SPG or PBS buffer or RPMI) were used as controls. In addition, chlamydial EB made non-infectious by heat inactivation was used as additional negative controls. 100 µl of Chlamydial EB were inactivated by heat treatment at 70°C for 30 min [28].

### Cytokine measurement

THP-1 cells were seeded in a 24-well plate at a cell number of 10^6^/well and infected with chlamydial EB at an MOI 2. After 4, 8, 16 and 24 hours post-infection, the cells were spun down and the supernatants were collected and stored at −80°C until further analysis. The supernatants collected from the cells were then analyzed for cytokines IL-1β, IL-6, TNF-α and IL-8 using ELISA kits (BD Biosciences). The assay was performed according to the manufacturer's instructions. THP-1 cells stimulated with 100 ng/ml *Escherichia coli* LPS (Sigma) for 4 h, and unstimulated THP-1 cells served as positive and negative controls, respectively.

### Measurement of surface receptor expression levels

THP-1 cells were seeded in a 24 well plate at a cell number of 10^6^/well and infected with chlamydial EB at an MOI 2. After 4, 8, 16 and 24 hours post-infection, the cells were spun down and fixed in 2% formaldehyde in PBS and adjusted to a final concentration of 5×10^5^/ml. After blocking in 2% BSA in PBS for 30 minutes, the cells were washed once with PBS and incubated with 10 µl of PE-conjugated mouse monoclonal anti-human ICAM-1 antibody per 10^6^ cells (eBiosciences) for 1 h. After staining, the cells were washed three times with PBS, and resuspended in 500 µl PBS. The samples were read in a flow cytometer (BD LSR II and BD FACS Diva software). Uninfected THP-1 cells labeled with PE-conjugated ICAM-1 antibody, and infected THP-1 cells labeled with isotype control served as negative controls.

### Endothelial cell receptor expression

Supernatant was collected from THP-1 cells infected for 16 h. The supernatant from uninfected or infected THP-1 cells were filtered using a 0.22 µm syringe filter to remove any chlamydial EB present in the solution. HAEC grown to confluence in 24-well plates was incubated with the filtered supernatant for 4 h. Then, the cells were gently scraped off the plate and were tagged with fluorescently labeled antibodies against VCAM-1, ICAM-1 or E-selectin (eBiosciences). The cells were analyzed by flow cytometry. HAEC exposed to uninfected, filtered supernatant was used as negative control.

### Exposure to shear stress

THP-1 cells were infected with chlamydial EB at MOI 2. 16 hours post-infection, the cells were spun down, and resuspended in RPMI complete medium containing 10 µg/ml gentamicin to a final concentration of 6×10^6^ cells/ml. 500 µl of this cell suspension was added to a clean, sterile cup of DVII+ Pro cone & plate viscometer (Brookefield Instruments) and sheared using a 1° cone for 20 minutes at a shear stress of 0 or 10 dyn/cm^2^. All the experiments were performed under sterile conditions. After shearing, the cells were counted, viability assessed using trypan blue stain, and adjusted to a final concentration of 10^6^ viable cells/ml in RPMI complete with 10 µg/ml gentamicin. The cells were then incubated for either 30 minutes or 3 h at 37°C in a humidified 5% CO_2_ incubator. After incubation, the cells were centrifuged, and the supernatant and cells was used for ELISA or flow cytometric assays, respectively.

### Platelet activation and aggregation

PRP was diluted in Dulbecco's PBS (DPBS) without calcium and magnesium (ATCC) to a concentration of 3×10^6^ platelets/10 µl. 10 µl of the platelet suspension was mixed with 90 µl filtered supernatant from infected THP-1 cells, and the mixture was immediately placed on the plate of cone-and-plate viscometer (Anton-Paar), maintained at 37°C. This mixture was sheared for 60 seconds at 0, 5 or 20 dyn/cm^2^. Immediately after shearing, 10 µl of platelets from the plate was drawn and fixed in 100 µl of 2% formaldehyde in DPBS. Non-specific adhesion was blocked with 100 µl of 2% BSA for 20 minutes. 10 µl of PE-conjugated mouse anti-human P-Selectin antibody (eBiosciences) was added per 10^6^ platelets, and incubated for 1 h. Staining was stopped with the addition of 800 µl of DPBS, and the platelets were analyzed for changes in mean fluorescence intensity (MFI), and shift in the forward scatter (FSC) channel, as measures of activation and aggregation respectively [29]. An increase in platelet cluster size due to aggregation results in an increase in the FSC read out, and was used as a measure of aggregation. Platelets treated with 100 µM ADP (Biodata) was used as positive control. Unstained and stained-unactivated platelet samples, and samples labeled with isotype antibodies were used as negative control.

### Perfusion assays

HAEC were cultured on 35 mm dishes to confluence, and was assembled to the bottom of a parallel plate flow chamber (Glycotech), separated by a thin silicone gasket. The thickness and width of the gasket determine the shear stress experienced by the HAEC monolayer due to fluid flow. The flow chamber was assembled on top an inverted microscope (Leica). Monocytes at a concentration of 10^6^/ml was drawn through the chamber using a syringe pump (Harvard apparatus) at a constant flow rate, corresponding to a shear stress of 1 dyn/cm^2^. The interaction between monocytes and the HAEC monolayer was observed at 400× magnification for four minutes in three different fields of view, and was recorded at 20 fps using a digital camera (Leica). The field of view was 0.2×0.2 mm^2^. The images were analyzed offline using NIH ImageJ software.

### Statistical analysis

Student's *t*-test was used to compare significant difference between different treatments. In order to assess the effect of infection and shear stress treatments compared to static, uninfected controls, one-way ANOVA was used. The differences were considered significant if the *P*<0.05.

## Results

### Chlamydial infection of monocytes

**Figure 1:**
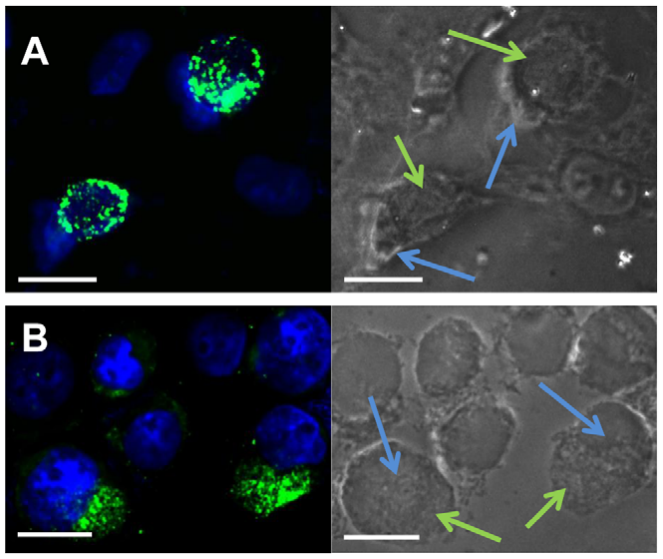
Chlamydial infection of mammalian cells. (A) Infection of adherent HeLa cells. HeLa cells were seeded at 10^6^/well into 24-well culture plates and infected with chlamydial EB at 10^5^ ifu/well for 24 h. The infected cells were fixed, and stained with rabbit anti-Chlamydia genus specific antibody followed by FITC-conjugated goat anti-rabbit IgG secondary antibody (green), and Hoechst nuclear stain (blue). The chlamydial inclusions (green) can be visualized next to the cell nuclei (blue) in infected cells. Some uninfected cells (nucleus only) also can be seen. Magnification: 250×. Scale bar is 20 µm; (B) Infection of suspension THP-1 monocytic cells. THP-1 cells infected at an MOI 2 for 24 hours were fixed and double stained with anti-chlamydia antibody (green) and Hoechst nuclear blue stain (blue). Chlamydial inclusions (green) and THP-1 cell nuclei (blue) are shown. Scale bar is 20 µm.

*Chlamydia muridarum*, being an intracellular bacterium, was cultured in a mammalian cell line. We used human epithelial cell line, HeLa to propagate *C. muridarum* following established protocol [30]. Chlamydia grows as an inclusion in the cytoplasm of the host cell. As the organism propagates, the inclusion grows over a period of 24 h, till the cell bursts and chlamydial EBs are released into the environment for infecting other host cells (Figure 1A). The HeLa cells were lysed after 24 h to collect EBs, and were used for infecting THP-1 monocytes.

The growth of chlamydial inclusion in suspension cultures of THP-1 monocytes followed a trend similar to that in the adherent HeLa cells. We observed the morphological changes by microscopy and noted that the chlamydial developmental lifecycle lasted ∼27 h in THP-1 monocytes, which is similar to that of HeLa cells. As the inclusion grows in size, the nucleus is displaced off-center accompanied by a modest increase in cell size. The increase in cell size was particularly noticeable in THP-1 cells (Figure 1B). The THP-1 cells infected with heat-inactivated chlamydial EB did not show any inclusion, indicating that heat-inactivated chlamydial EB are non-infective (Figure S1).

### Cytokine release from infected monocytes

**Figure 2:**
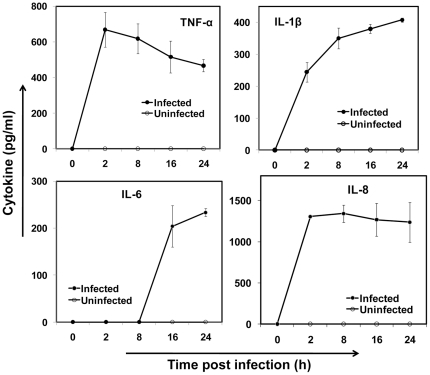
Cytokine release from infected monocytes. THP-1 monocytes were infected with mock PBS or chlamydial EB (MOI 2) for 2 h and cultured for 24 h. At 2, 8, 16 and 24 h, the supernatant was analyzed by ELISA for TNF-α, IL-1β, IL-6 and IL-8. The results are mean ± SD of a representative experiment performed in triplicate, and each experiment was repeated at least three times.

Upon chlamydial infection, cells of the vasculature including endothelial, smooth muscle cells [31], and leukocytes [32], [33] secrete pro-inflammatory cytokines. The cytokines TNF-α, IL-1β IL-6, and IL-8 are important in vascular inflammation and atherosclerosis. The pro-inflammatory cytokines TNF-α, IL-1β, IL-6, and other acute phase proteins such as serum amyloid A (SAA) and C-reactive protein (CRP) have been associated with atherosclerosis or increased risk of cardiovascular disease [34]. IL-8 is a CXC chemokine that triggers monocyte recruitment to the site of infection or injury during vascular inflammation [35]. Hence, we measured the release of the cytokines TNF-α, IL-1β, and IL-6, and chemokine IL-8 from infected monocytes using ELISA. As shown in Figure 2, all the cytokine levels increase over the 24 h period. The acute phase cytokines, TNF-α and IL-1βand chemokine IL-8 levels increase within 2 h following infection, while IL-6 expressed late, at 8 to 16 h post infection. Cytokine released from uninfected monocytes were below detectable limits.

### Effect of shear stress on cytokine release from infected monocytes

**Figure 3:**
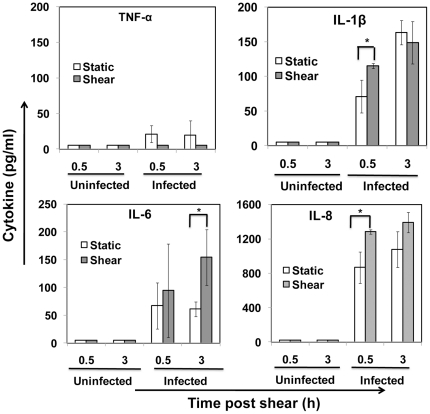
Effect of shear stress on cytokine release from infected monocytes. THP-1 monocytes were infected with mock PBS or chlamydial EB (MOI 2) for 16 h. 10^6^ uninfected and infected cells were sheared for 20 min at 0 (static) or 10 dyn/cm^2^ (shear) using a cone-and-plate viscometer, and were incubated for either 30 min or 3 h post shear. The supernatants were analyzed by ELISA for cytokines TNF-α, IL-1β, IL-6, and IL-8. The results are expressed as mean ± SD of one representative experiment performed in triplicate, and the experiments were performed three times. The * denote statistically significant increase (*P*<0.05, Student's *t*-test) in cytokine release due to shear stress of infected cells compared to unsheared, static treatment.

Chlamydial infection of host cells including those of epithelial cells in the lung or genital tract activates the rapid recruitment of phagocytic cells. The phagocytic cells such as neutrophils and peripheral blood mononuclear cells (PBMC) ingest chlamydia, and thus act as vehicles for systemic dissemination to distant sites [17]. *C. pneumoniae* has been shown to be transported to sites of atherosclerotic plaques by monocytes. As the infected monocytes travel through circulation, they experience hydrodynamic shear stress due to blood flow. We are interested in evaluating how the shear stress may influence the response of chlamydia-infected monocytes. To this end, we subjected monocytes infected for 16 h to arterial shear stress of 10 dyn/cm^2^ for 10 min, and analyzed cytokine expression at 30 min and 3 h post shear. We chose 16 h post infection as a representative time point because all the cytokines are expressed by 16 h (Figure 2), and the inclusions are sufficiently large when viewed under a microscope (data not shown). We chose to measure freshly expressed cytokine levels at 30 min and 3 h post-shear so as to capture both spontaneous release of already synthesized cytokines and that of cytokines synthesized *de novo*, due to shear stress exposure. The exposure of cells to shear stress did not result in any appreciable decrease in viability as measured by trypan blue assay (Figure S2). As shown in Figure 3, we observed that 10 dyn/cm^2^ shear stress elicited varied responses in the four cytokines: (a) TNF-α level was undetectable, indicating there was no new TNF-α released after 16 h of infection due to shear stimulation; (b) IL-1β and IL-8 was modestly upregulated due to shear stress at 30 min, but this difference became insignificant at 3 h; and (c) IL-6 level was upregulated due to shear stress by more than 100% at 3 h, but not within 30 min, post-shear. As positive control, monocytes stimulated with LPS released appreciable quantities of all four cytokines TNF-α, IL-1β, IL-6 and IL-8, measured by ELISA (data not shown).

### Cytokine-mediated platelet activation and aggregation under shear

**Figure 4:**
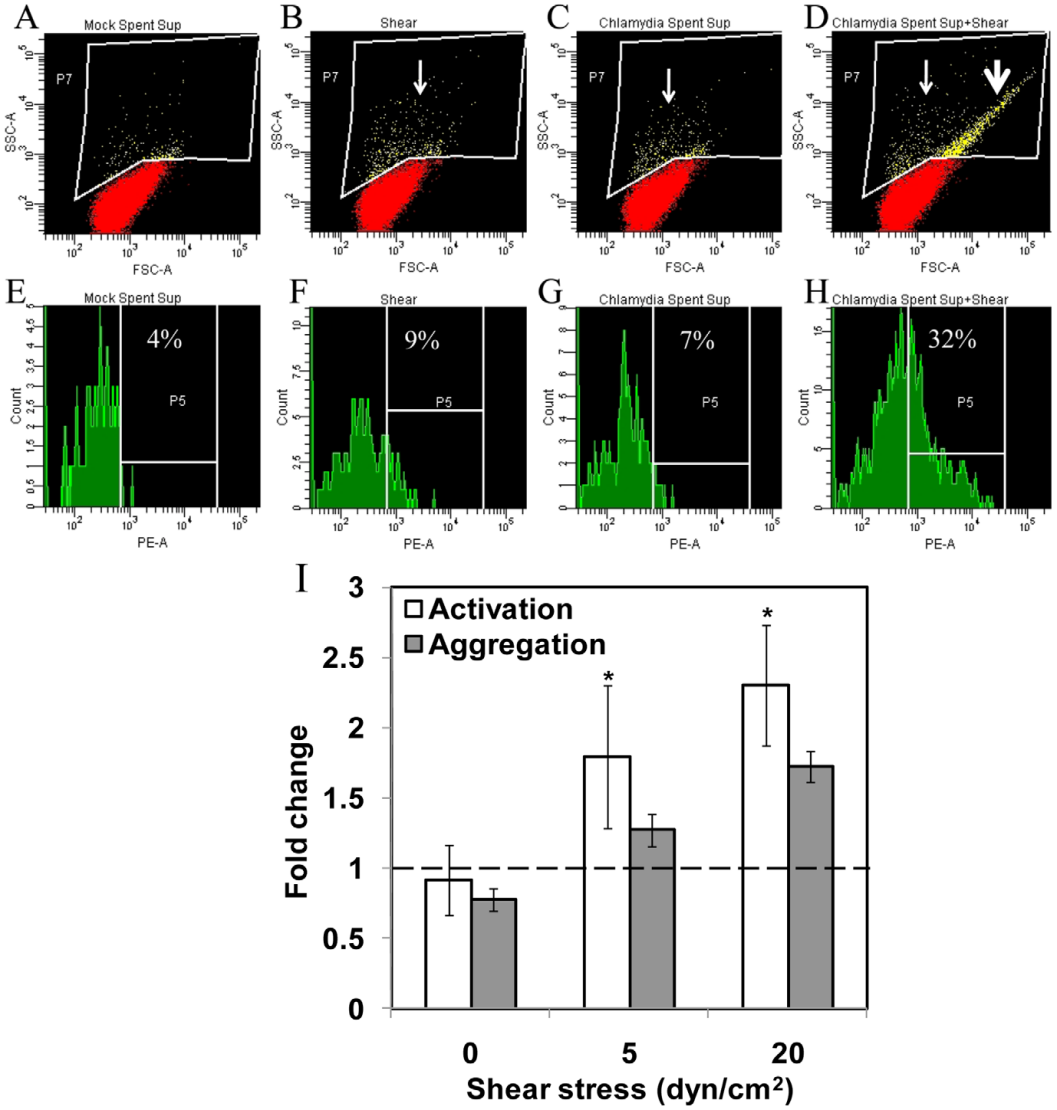
Effect of shear stress and infection on platelet activation and aggregation. Supernatant from 16 h infected or uninfected THP-1 monocytes were added to freshly isolated human platelets. After 90 s, the platelets were subjected to a shear stress of 0, 5 or 20 dyn/cm^2^ for 60 s. Platelets were fixed, and stained with PE-conjugated anti-CD62P (P-Selectin) antibody to measure activation (**A–D**), and aggregation (**E–H**). Platelets that demonstrated enhanced granularity (thin arrow), and enhanced size/aggregation and granularity (thick arrow) were included into the gate, and were further analyzed for the presence of anti-CD62P staining. The results are expressed as percentage of CD62P positive events within the gate drawn around platelets displaying enhanced size and/or granularity. (**I**) Ratio of activated or aggregated platelets upon treatment with infected spent supernatant to that due to exposure to mock supernatant was calculated. The results are mean ± SD of experiments performed in duplicate with blood drawn from two different volunteers. The * denote statistically significant increase (*P*<0.05, Student's *t*-test) in platelet aggregation and activation due to shear stress compared to unsheared, static treatment (denoted by dotted line).

Platelet activation and aggregation are both an important cause and consequence of vascular inflammation. Activated platelets are identifiable in the circulation of patients with diabetes and atherosclerosis, and in septic shock [36]. We hypothesized that the cytokines released from chlamydia-infected monocytes in circulation can activate and aggregate platelets. We exposed freshly isolated to human platelets to supernatant obtained from monocytes infected for 16 h under venous (5 dyn/cm^2^) and arterial (20 dyn/cm^2^) shear conditions using a cone-and-plate viscometer, and measured platelet activation and aggregation (Figure 4A–H). P-selectin expression on platelet surface was used as a marker for platelet activation. We observed no spontaneous activation, when platelets were exposed to supernatant collected from uninfected or infected monocytes in the absence of shear, i.e., static conditions. We also observed that exposure of platelets to shear stress alone did not result in the activation of platelets. However, when platelets were exposed to supernatant collected from infected monocytes at an arterial shear stress of 20 dyn/cm^2^ for 60 s, we observed a significantly enhanced P-selectin expression as evidenced by shift in the platelet population in the forward/side scatter plot of the flow cytometer. Shear stress also significantly enhanced aggregation of platelets that are exposed to supernatant from chlamydia-infected monocytes. The increase in platelet activation and aggregation were quantified as fraction of the total population from the flow cytometric analysis (Figure 4I). We used platelet activation and aggregation under static conditions as reference. Under static conditions, small platelet aggregates disaggregated resulting in a mild decrease in platelet aggregation. When exposed to shear, we observed that platelet activation and aggregation increased in a shear-dependent manner compared to static control.

### Endothelial activation and monocyte adhesion under flow

**Figure 5:**
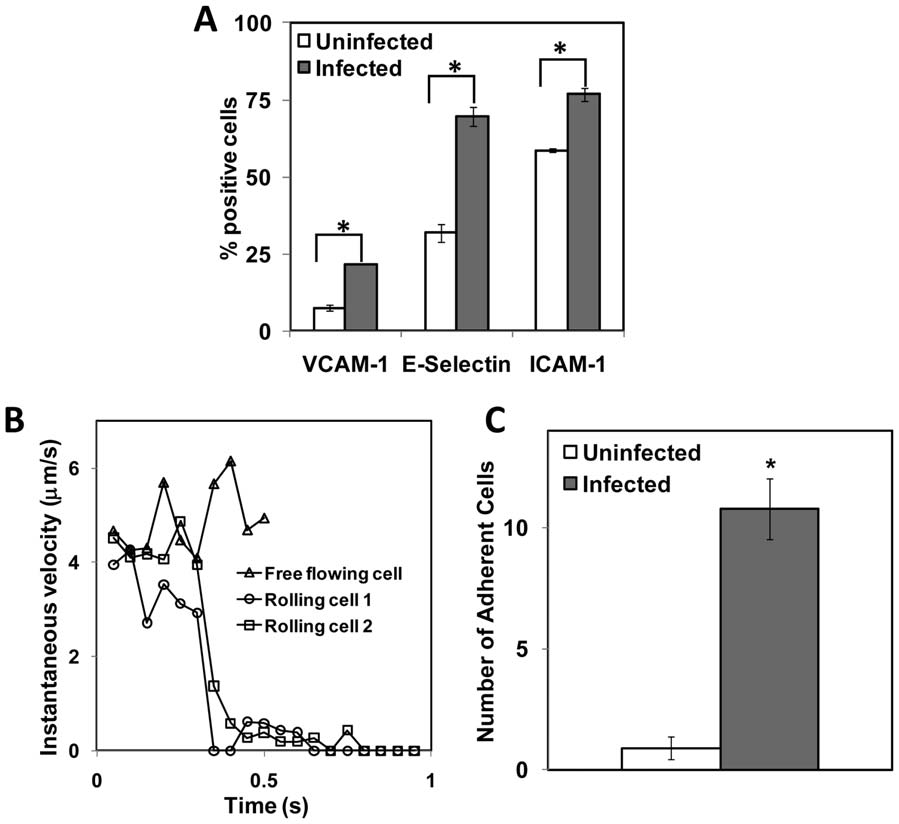
Infected monocyte supernatant activates endothelial cells and support monocyte adhesion under shear. HAECs were activated with either supernatant collected from 16 h chlamydia-infected monocyte or mock (negative control) or 100 nM TNF-α (positive control). (A) HAEC were fixed, stained with appropriate antibodies and analyzed by flow cytometry. The results are average of a representative experiment run in triplicate, and the experiment was repeated two times. Isotype control showed basal activity, and TNF-α control showed positive expression of all markers (data not shown). The * denote statistically significant increase (*P*<0.05, Student's *t*-test) of treatment compared to mock. (B) Uninfected monocytes were perfused on top of HAEC activated with supernatant from infected monocytes. The interaction between monocyte and endothelial cells were recorded, and the rolling velocity was calculated from each frame off-line. Some cells get captured to the surface, rolled for a short distance before adhering firmly to the surface. (C) The number of adherent cells was calculated at four different fields of view, and the results shown are mean ± SD of triplicates of one experiment, and the experiment was performed three times. The * denote statistically significant increase (*P*<0.05, Student's *t*-test) in the number of adherent monocytes due to infection compared to uninfected control.

During inflammation due to injury or an infection, the vascular endothelium gets activated with substantial changes to the endothelial phenotype, thus converting a passive surface to a pro-cell adhesive phenotype [10]. The adhesive endothelium recruits leukocytes from flowing blood to the vessel wall to resolve the injury or infection, as a normal inflammatory response. However, if the inflammatory process extends over long periods of time, it can result in excessive tissue injury and chronic diseases such as atherosclerosis. *C. pneumoniae* has been isolated from atherosclerotic plaques, but not normal arterial walls, and is believed to have been transmitted by *C. pneumoniae*-infected monocytes, which adhere to and transmigrate through the endothelium into the tunica media. The presence of *C. pneumoniae*-infected monocytes in the arterial wall can activate the neighboring endothelium leading to further monocyte recruitment and the formation of atherosclerotic plaques. We studied the effect of chlamydial infection on monocyte adhesion to endothelium under flow conditions. First, we analyzed the activation of cultured human aortic endothelial cells (HAEC) exposed to supernatant from monocytes infected for 16 h. We measured the expression of surface adhesion molecules, E-selectin, ICAM-1 and VCAM-1 (Figure 5A). We observed that all the adhesion molecules were significantly upregulated by infected monocyte supernatant compared to uninfected monocyte supernatant. E-selectin and VCAM-1 expressions increased by ∼100% due to infection, and ICAM-1 expression showed a moderate increase. We observed that the basal ICAM-1 level expression was relatively high in HAEC, consistent with published reports [37].

In order to study the effect of chlamydial infection of endothelial cells in mediating monocyte adhesion under shear conditions, we perfused THP-1 monocytes at 1 dyn/cm^2^, over a monolayer of HAEC that were exposed to infected or uninfected monocyte supernatant. Monocytes tethered on to the endothelium, rolled briefly before adhering firmly (Figure 5B). We observed that a large number of monocytes adhered to HAEC that are exposed to monocyte supernatant, while almost none adhered to untreated HAEC (Figure 5C).

**Figure 6:**
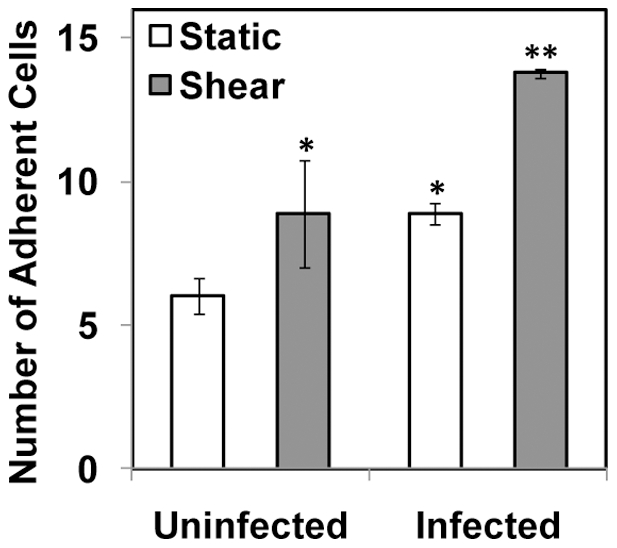
Monocyte adhesion to endothelial cells activated with supernatant from infected and sheared monocytes. THP-1 monocytes were infected with mock PBS or chlamydial EB (MOI 2) for 16 h. Uninfected and infected cells were sheared for 20 min at 0 (static) or 10 dyn/cm^2^ (shear) using a cone-and-plate viscometer, and the supernatant was used to activate HAEC. Uninfected monocytes were perfused on top of HAEC, and the number of adherent cells was calculated in three different fields of view. The results are mean ± SD of one representative experiment performed in triplicate, and the experiments were repeated three times. The * denote statistically significant increase in the number of adherent monocytes due to shear stress or infection compared to uninfected, static control; and ** denotes statistically significant increase due to both shear stress and infection compared to all other groups (*P*<0.05, ANOVA).

We have observed that shear stress considerably increases the expression of certain cytokines from infected monocytes (Figure 3). When the infected monocytes are in close proximity to the endothelium such as when the infected monocytes are lodged in the subendothelial matrix, it can be expected that these cytokines can activate the endothelium leading to the recruitment of monocytes from circulation. In order to evaluate the effect of increased cytokine levels due to shear stress and infection on endothelial cells, we perfused uninfected monocytes on endothelial cells treated with the supernatant collected from chlamydia-infected monocytes that were exposed to 0 or 10 dyn/cm^2^ shear stress for 20 min. We observed while shear stress or infection alone showed a modest increase in the number of adherent monocytes, shear stress and infection together increased the number of monocytes adhering to the endothelium (Figure 6).

**Figure 7:**
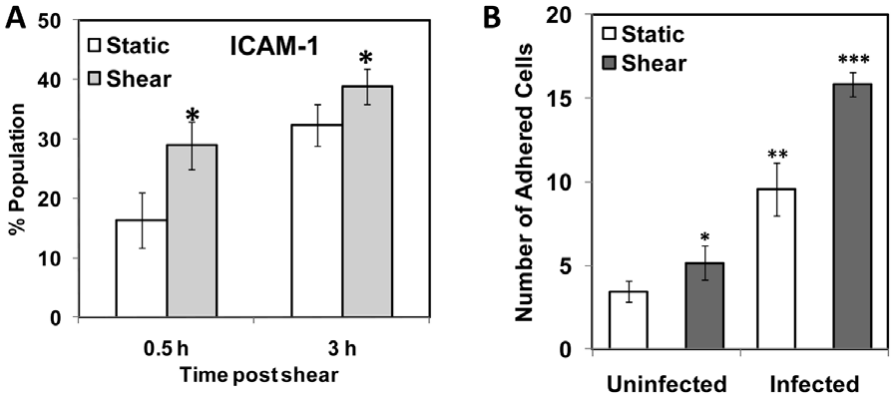
Effect of shear stress on ICAM-1 expression and endothelial adhesion of infected monocytes. (A) THP-1 monocytes were infected with mock PBS or chlamydial EB (MOI 2) for 16 h. Uninfected and infected cells were sheared for 20 min at 0 (static) or 10 (shear) dyn/cm^2^ using a cone-and-plate viscometer, and were incubated for either 30 min or 3 h post shear. The cells were fixed, stained with FITC-conjugated ICAM-1 antibody and analyzed by flow cytometry. The * denote statistically significant increase (*P*<0.05, Student's *t*-test) in ICAM-1 expression due to shear; (B) HAECs were activated with 100 nM TNF-α and assembled in a parallel plate flow chamber. Infected monocytes were sheared either at 0 (static) or 10 (shear) dyn/cm^2^ for 20 min, incubated for 30 min, and then perfused through the flow chamber at 1 dyn/cm^2^. The number of adherent cells was counted from four different fields of view. The results are mean ± SD of one representative experiment performed in triplicate, and the experiments were repeated three times. The *, **, and *** denote statistically significant increase in the number of infected, monocytes adhered to HAEC between different groups (*P*<0.05, ANOVA).

In addition to the effect of cytokines from infected monocytes, we analyzed the effect of shear stress on the infected monocytes themselves. We noticed that shear stress increases the upregulation of adhesion molecule ICAM-1 (Figure 7A). ICAM-1 is an important surface receptor in the immunoglobulin family, which mediates firm adhesion of monocytes to endothelium. We perfused monocytes infected for 16 h and sheared at 10 dyn/cm^2^ for 20 min, on top of HAEC activated with TNF-α at a shear stress of 1 dyn/cm^2^, and the number of adherent monocytes was counted. As shown in Figure 7B, infected or sheared monocytes adhered to the endothelium more than uninfected and unsheared monocytes, and infected monocytes adhered more than sheared monocytes. In contrast, subjecting infected monocytes to short duration of arterial shear stress significantly increased their adhesion to activated endothelium. This suggests a synergistic interaction between shear stress and infection on monocyte adhesion to endothelium.

## Discussion

In this study, we have demonstrated that hydrodynamic shear stress modulates the inflammatory response of monocytes following chlamydial infection. We found that physiological levels of shear stress for a short duration significantly upregulated the expression of pro-inflammatory cytokines in infected monocytes. We also found that shear stress enhanced the adhesion of infected monocytes to activated endothelium under flow conditions. Shear stress is also an important mediator in activating and aggregating platelets in the presence of cytokines released from infected monocytes. Our results, thus demonstrate that, fluid mechanical forces experienced by infected cells in the vasculature can be a significant determinant in the overall inflammatory response. These observations have long-reaching impact in our understanding of systemic infections, which have been studied predominantly under static conditions. Our results underscore the importance of mechanical forces as potent regulators of cellular and molecular mechanisms of infectious diseases, which heretofore has not been recognized.

Chlamydiae are unique among the intracellular pathogens in that they maintain a large, stable, membranous vacuole, which is central to the developmental cycle. Consistent with previous reports, we noted that these inclusions push the nucleus to one side of the cell [38]. We observed such a nuclear displacement, accompanied by an increase in cell size, by a growing inclusion both in the adherent epithelial cell and suspension monocyte cultures. This alteration may influence cellular transport and cell adhesion processes. Infection of epithelial cells with *C. trachomatis* and *C. pneumoniae* has been shown to cause a substantial cytoskeletal rearrangement [38].

Infection of human monocytes by *C. pneumoniae* has been reported to result in the upregulation of cytokines and chemokines [39], [40]. Stimulation of human PBMC with *C. pneumoniae* induces the production of chemokines IL-8, MIP-1α and MCP-1, and cytokines TNF-α, IL-1β and IL-6 [32]. *C. pneumoniae* induced the production of TNF-α, IL-10 and IL-12 in THP-1 monocytic cell line [39], and gene expression of a number of cytokines including IL-1β, TNF-α, MCP1, MIP-1α in a time-dependent fashion in U937 monocytic cell line [40]. Consistent with these reports, we observed a time-dependent increase in the expression of TNF-α, IL-1β, IL-6 and IL-8. We also observed that non-infective heat-inactivated chlamydial EB also elicited a cytokine response similar to infective, live organisms suggesting that dead chlamydial organisms may contribute to inflammatory processes (Figure S3) [32]. These experiments were done exclusively under static conditions, wherein the cytokines released by the host cell due to chlamydial infection cultured in the absence of any external force fields were analyzed in the supernatant. However, *in vivo*, as the infected monocytes travel through circulation, they experience hydrodynamic shear stress. It is now well documented that fluid shear stress due to blood flow influences cellular and molecular mechanisms of inflammation [6]. In this study, we have demonstrated that physiological levels of shear stress on chlamydia-infected monocytes modulate the release of previously synthesized cytokines or triggers *de novo* synthesis of fresh cytokines. We observed that no TNF-α was secreted into the media 16 h post-infection under both static and shear conditions. TNF-α is an acute phase cytokine, which is released immediately following an infection. Hence, it is possible that all the TNF-α reserves are exhausted in 16 h post-infection, and no fresh TNF-α is synthesized and released from monocytes in response to shear stress. On the other hand, monocytes secrete more IL-8 and IL-1β immediately after exposure to shear stress compared to static control, and this difference vanishes at 3 h post shear. IL-8 is an inflammatory chemokine stored in the Weibel-Palade bodies of monocytes, platelets and endothelial cells, and thus are immediately released upon shear activation of infected monocytes. IL-1β is generated from cytoplasmic pro-IL-1β, which is cleaved by caspase-1 in response to infection, inflammation or immunological challenges [41]. Our data that IL-1β is overexpressed shortly after exposure to shear stress suggests that pro-IL-1β cleavage by caspase-1 may be stimulated by mechanoreceptors on cell surface. One possible signaling pathway is the activation of transcription factor Nf-κB by mechanical forces, which in turn activates caspase-1, leading to pro-IL-1β cleavage. In contrast, IL-6 is significantly upregulated (more than 100%) not immediately but shortly after shear exposure. Thus, fresh IL-6 is synthesized *de novo* in response to shear stress, and is controlled at transcriptional level. In essence, shear stress affects cytokine release both at the transcriptional and cytosolic levels from infected monocytes. However, we observed that exposure of monocytes to short durations of shear stress alone did not elicit any cytokine release from uninfected cells. Thus, the profound effect of shear stress on infected, but not uninfected, monocytes clearly point to the importance of synergistic interaction between biochemical and biomechanical signals in determining inflammatory response.

Once produced, the cytokines stimulate neighboring cardiovascular cells to modulate an array of cellular functions thus contributing substantially to vascular inflammation [42]. We were interested in the effect of cytokines released by infected monocytes on other vascular cells including platelets and endothelium in the presence of hydrodynamic shear stress. Platelets in circulation are activated by systemic thromboembolic or inflammatory events, and activated platelets have been implicated in thrombosis, inflammatory reactions, immune responses, and in distinct aspects of atherosclerosis [43]. Since chlamydia-infected monocytes continuously release cytokines, we evaluated platelet activation through P-selectin upregulation and platelet aggregation due to chlamydial infection of monocytes in circulation. We observed that both infected monocyte supernatant and shear stress, in combination but not individually, resulted in significant platelet activation and aggregation. Platelets do not get activated spontaneously at physiological levels of shear stress such as those found in arteries and veins. High shear stress, such as those found in stenotic vessels spontaneously activates and aggregates platelets [44]. Thus, our observation implies that systemic chlamydial infection can be as potent a stimulus as high shear stress in activating platelets. As shown in Figure 2, the infected monocyte supernatant contain cytokines including the chemokine IL-8, is an activator of platelets. The monocyte supernatant may also contain other molecules such as platelet activating factors, which is not measured in this study. It should also be noted that the secretions from infected monocytes themselves do not result in platelet activation under static conditions, emphasizing the interplay between biochemical and biomechanical signaling in systemic infection and inflammation.

The sustained presence of inflammatory mediators in the vasculature can lead to vessel wall endothelial activation. As shown in Figure 2, infected monocytes continuously secrete cytokines, which when presented to vessel wall endothelium, can activate the endothelial cells. At least two instances that can conceivably result in high cytokine levels in endothelial microenvironment due to monocyte – endothelial proximity are when infected monocyte from flowing blood adheres to the endothelium; and when an infected monocyte is lodged in the vascular wall matrix underneath the endothelium. Endothelial cell activation is characterized by the upregulation of a number of adhesion receptors of the immunoglobulin superfamily including intercellular adhesion molecules, and vascular cell adhesion molecules, and the carbohydrate binding selectins. We observed that ICAM-1, VCAM-1 and E-selectin are significantly upregulated when endothelial cells were exposed to supernatant from infected monocytes. We also observed that under flow conditions, the monocytes adhered to the endothelium, which were previously treated with filtered supernatant from infected monocytes. The supernatant is rich in cytokines TNF-α, IL-8, IL-6, and IL-1β (Figure 2), which are known to be potent activators of endothelium. The adhesion of unactivated monocytes to endothelium is presumably mediated by receptors upregulated on endothelial cells that are treated with supernatant from infected monocytes. Endothelial E-selectin and VCAM-1 mediate transient interactions such as monocyte tethering and rolling by binding to their cognate ligands, E-selectin ligand-1 (ESL-1) and VLA-4, respectively on the monocyte surface. Endothelial ICAM-1 mediates firm arrest of rolling monocytes by binding to integrins LFA-1 and Mac-1 [45].

After the capture of monocytes on to activated endothelium, monocytes adhere firmly and transmigrate into the arterial wall. ICAM-1 is an important mediator of firm adhesion and transmigration of monocytes through the endothelium [45]. ICAM-1 is significantly upregulated in infected monocytes exposed to arterial shear stresses for a short duration, compared to static control (Figure 7). When perfused through a parallel plate flow chamber, this increase in ICAM-1 expression in infected and sheared monocytes results in increased adhesion to TNF-α-activated HAEC, compared to either infected or sheared monocytes alone. Taken together, the results in Figures 6 and 7 show that infected and sheared monocytes adhere to endothelial cells are activated either by recombinant TNF-α, or cytokines released from infected monocytes, under flow conditions. These results point to a possibly an important role of *Chlamydia pneumoniae* in early stages of atherosclerosis. *C. pneumoniae* infects alveolar macrophages and epithelial cells in the lung, and is transmitted to peripheral blood monocytes, which serve to disseminate the organism to distant sites [17]. As the infected monocytes travel through the circulation, they may exhibit a greater propensity to adhere to endothelial cells and extravasate. Once the infected monocytes lodge in the subendothelium, continued release of cytokines can activate the neighboring endothelial cells, which may recruit fresh monocytes from the circulation.

Our finding that fluid shear stress can exacerbate the inflammatory response due to an intracellular pathogen leads us to some speculative remarks concerning the physiological significance of hydrodynamic regulation of infection. Based on our results that fluid shear stress can modulate the inflammatory response due to an infection, we are tempted to suggest that mechanical forces due to blood flow may play a critical role in the progression of diseases caused by intracellular pathogens such *Mycobacterium tuberculosis* or extracellular pathogens such as *Staphylococcus aureus*, which infect the cells in the vasculature. Currently available literature on infection and inflammation has exclusively focused on static cultures. We show that mechanical forces play an important role in modulating the cellular response to infection and may determine disease pathophysiology. Thus, our study underscores the importance of and provides a framework to elucidate the role of mechanical signals on infection, inflammation, and pathogenesis of human disease.

## Supporting Information

Figure S1 Chlamydial infection of monocytes. THP-1 monocytes were infected with (A) mock buffer (SPG or PBS), (B) live, or (C) heat-inactivated chlamydial EB (MOI 2) for 24 hours and stained with rabbit anti-Chlamydia genus specific antibody followed by FITC-conjugated goat anti-rabbit IgG secondary antibody (green), and Hoechst nuclear stain (blue). The chlamydial inclusions (green, arrow) can be visualized next to the cell nuclei (blue) only in cells infected live chlamydial EB and not with heat-inactivated EB. The scale bar is 10 µm.
(0.68 MB TIF)

Figure S2 Viability of infected and sheared monocytes. THP-1 monocytes were infected with mock PBS or chlamydial EB (MOI 2) for 16 hours. 10^6^ uninfected and infected cells were sheared for 20 minutes at 0 (static) or 10 dyn/cm^2^ (shear) using a cone-and-plate viscometer and were incubated for either 30 minutes or 3 hours post shear. The cells were then tested for viability using trypan blue staining. The results are expressed as mean ± SD of one representative experiment performed in triplicate, and the experiments were performed three times. Shear did not significantly affect the viability of either infected or uninfected cells.
(0.07 MB TIF)

Figure S3 Cytokine release from infected monocytes. THP-1 monocytes were infected with mock PBS or SPG buffer, or heat-inactivated or active chlamydial EB (MOI 2) for 2 hours and cultured for 16 hours. The supernatants were collected at 16 hours and were analyzed by ELISA for TNF-α, IL-1β, IL-6 and IL-8. The results are mean ± SD of one experiment performed in triplicate and the experiments were performed two times. The cytokine expression levels were similar in THP-1 cells infected with live and dead organisms.
(0.07 MB TIF)
