# Supplementary figures and images for: Hydrodynamic Regulation of Monocyte Inflammatory Response to an Intracellular Pathogen
Source: PLoS One. 2011 Jan 7;6(1):e14492. doi: 10.1371/journal.pone.0014492 (PMC3017540; doi:10.1371/journal.pone.0014492)

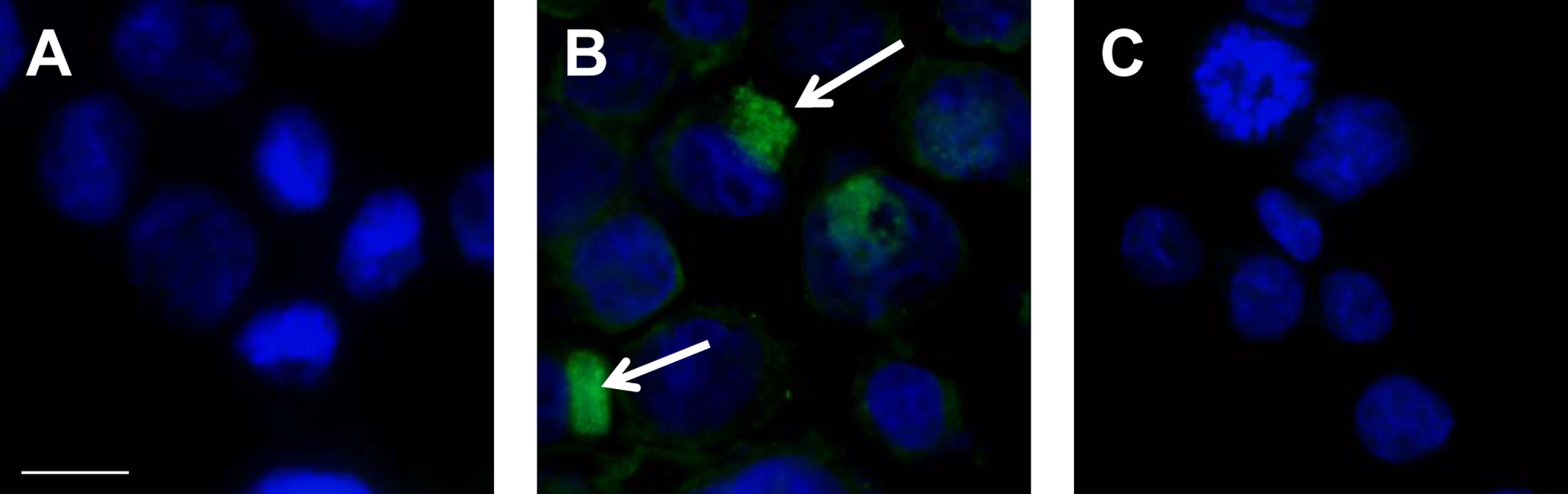

Supplement: Figure S1 — Chlamydial infection of monocytes. THP-1 monocytes were infected with (A) mock buffer (SPG or PBS), (B) live, or (C) heat-inactivated chlamydial EB (MOI 2) for 24 hours and stained with rabbit anti-Chlamydia genus specific antibody followed by FITC-conjugated goat anti-rabbit IgG secondary antibody (green), and Hoechst nuclear stain (blue). The chlamydial inclusions (green, arrow) can be visualized next to the cell nuclei (blue) only in cells infected live chlamydial EB and not with heat-inactivated EB. The scale bar is 10 µm. (0.68 MB TIF) [file pone.0014492.s001.tif]

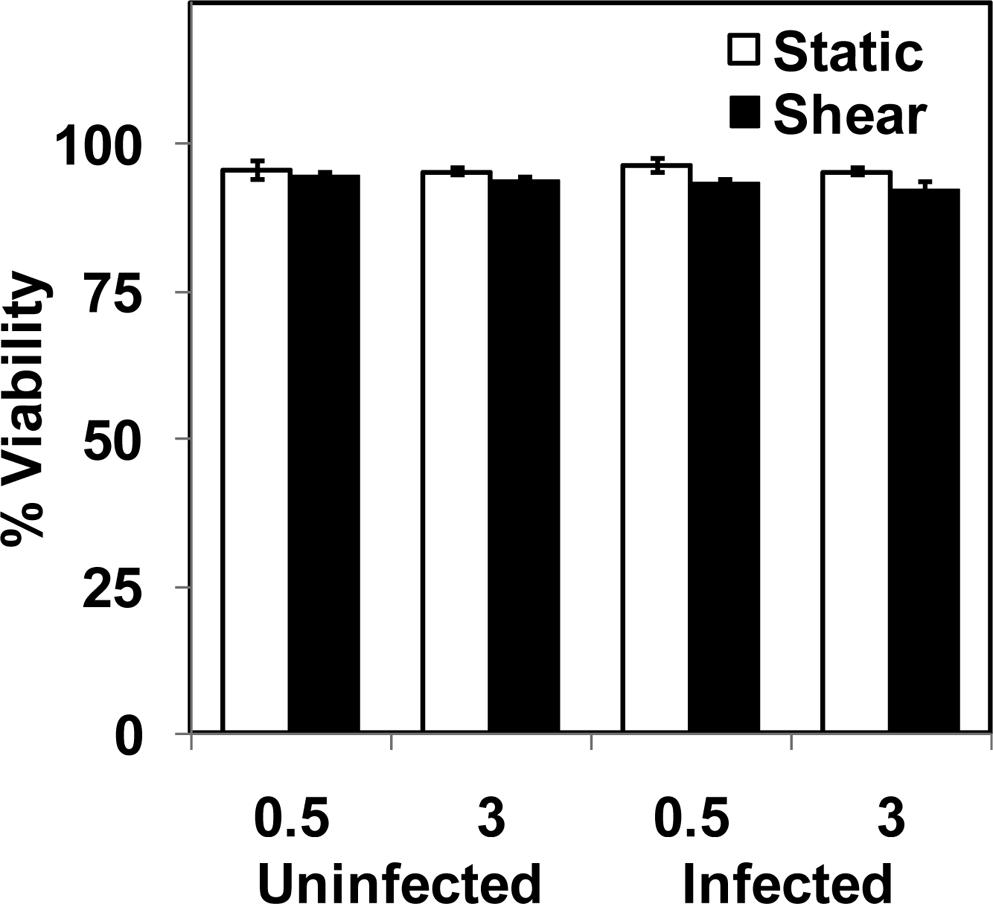

Supplement: Figure S2 — Viability of infected and sheared monocytes. THP-1 monocytes were infected with mock PBS or chlamydial EB (MOI 2) for 16 hours. 106 uninfected and infected cells were sheared for 20 minutes at 0 (static) or 10 dyn/cm2 (shear) using a cone-and-plate viscometer and were incubated for either 30 minutes or 3 hours post shear. The cells were then tested for viability using trypan blue staining. The results are expressed as mean ± SD of one representative experiment performed in triplicate, and the experiments were performed three times. Shear did not significantly affect the viability of either infected or uninfected cells. (0.07 MB TIF) [file pone.0014492.s002.tif]

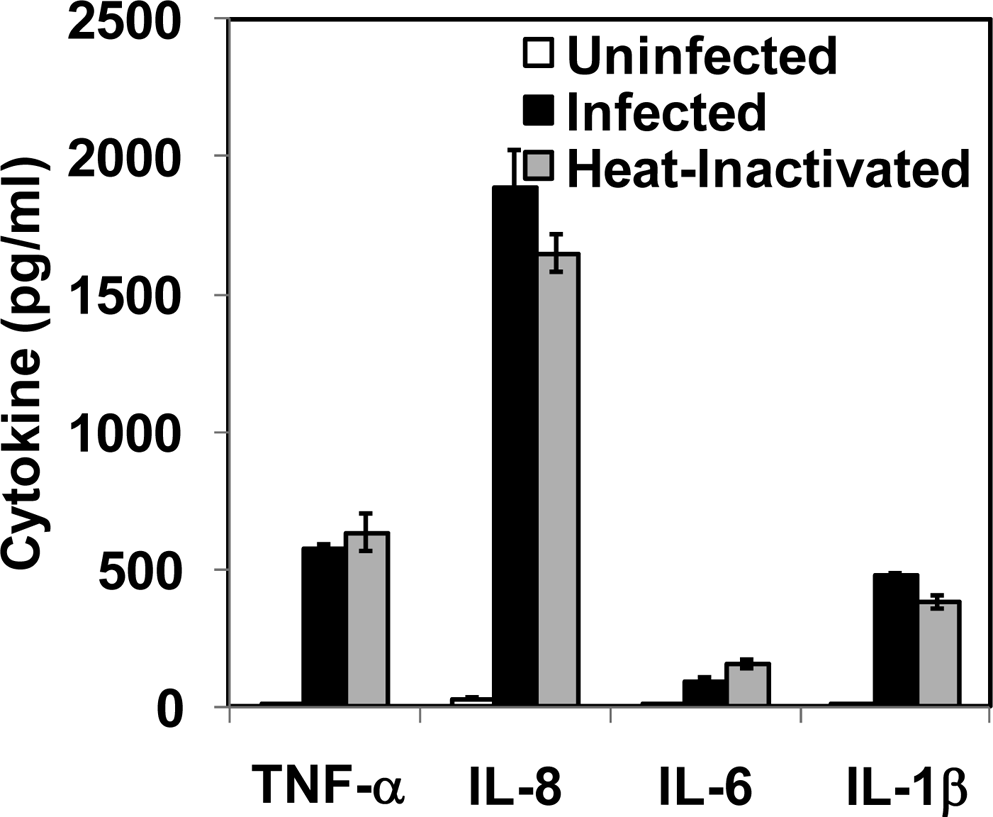

Supplement: Figure S3 — Cytokine release from infected monocytes. THP-1 monocytes were infected with mock PBS or SPG buffer, or heat-inactivated or active chlamydial EB (MOI 2) for 2 hours and cultured for 16 hours. The supernatants were collected at 16 hours and were analyzed by ELISA for TNF-α, IL-1β, IL-6 and IL-8. The results are mean ± SD of one experiment performed in triplicate and the experiments were performed two times. The cytokine expression levels were similar in THP-1 cells infected with live and dead organisms. (0.07 MB TIF) [file pone.0014492.s003.tif]
